# Milk Performance and Grazing Behaviour of Cinisara Cows Supplemented with Low- and High-Polyphenols Faba Bean Varieties

**DOI:** 10.3390/ani15030335

**Published:** 2025-01-24

**Authors:** Massimiliano Lanza, Marialetizia Ponte, Marianna Pipi, Adriana Bonanno, Antonino Di Grigoli, Marcella Avondo, Serena Tumino

**Affiliations:** 1 Dipartimento di Agricoltura, Alimentazione e Ambiente, University of Catania, 95123 Catania, Italy; m.lanza@unict.it (M.L.); serena.tumino@unict.it (S.T.); 2 Dipartimento di Scienze Agrarie, Alimentari e Forestali, University of Palermo, 90128 Palermo, Italy; marialetizia.ponte@unipa.it (M.P.); marianna.pipi@unipa.it (M.P.); adriana.bonanno@unipa.it (A.B.); antonino.digrigoli@unipa.it (A.D.G.)

**Keywords:** faba bean supplement, polyphenols, grazing cows, milk

## Abstract

The high degradability of crude protein (CP) from fresh grass can impair its utilization in ruminants. The presence of a moderate level of polyphenolic substances in the diet of grazing animals could help to overcome this problem. The study aimed to evaluate the effects of supplementation with two varieties of faba bean, with different polyphenol contents, on milk yield and quality, as well as on grazing behaviour, in Cinisara grazing cows. A total of 30 cows, homogeneous per days in milk (61 ± 29) and milk yield (12.9 kg ± 3.2), fed on a mixed pasture (CP 17.9% DM) and supplied with grains (an equal mixture of barley, oats, and wheat) and mixed hay (CP 10.2% DM), were assigned to three dietary treatments and supplemented as follows: control group (C, 10 cows), 1 kg of hay, 6 kg of grains mixture, and 0.4 kg soybean meal; low polyphenols group (LP, 10 cows), 1 kg of hay, 4 kg of grains mixture, and 2 kg/d of faba bean var. Torrelama (total polyphenols 4.4 mg GAE/g DM; CP 28.4% DM); high polyphenols group (HP, 10 cows), 2 kg of hay, 4 kg of grains mixture, and 2 kg/d of faba bean var. Fanfare (total polyphenols 16.4 mg GAE/g DM; CP 28.9% DM). All groups were allowed to graze for 20 h/d on natural pasture forage. The HP supplement tended to increase the milk yield compared to that of the LP and C groups (17.1 vs. 15.3 and 14.6 kg/d, respectively; *p* = 0.057) but reduced the protein (3.20 vs. 3.39 and 3.47%; *p* = 0.009) and casein proportions (2.45 vs. 2.67 and 2.74%, respectively; *p* = 0.007) compared to those from the LP and C treatments. HP milk also showed a higher milk urea nitrogen (MUN) value compared to that of C milk, while an intermediate level was measured in LP milk (25.5 vs. 22.9 and 20.9 mg/dl, respectively; *p* = 0.036). No dietary effect was evident in the milk fatty acid profile. Eating time at pasture and biting rate were not affected by supplementation. The results do not seem to suggest a difference in the efficiency of use of supplements with different polyphenol contents. In any case, they seem to demonstrate that the use of faba beans, regardless of their polyphenol content, represents a valid alternative to soyabeans, also taking into account the tendency for an increased milk yield found by integrating pasture grazing with faba beans possessing a high level of polyphenols, without significant worsening of the qualitative characteristics of the milk or negatively affecting grazing behaviour.

## Simple Summary

One of the great challenges regarding pasture-grazing cows is an often significant intake of early-maturing herbage rich in highly soluble protein that could impair rumen efficiency. One of the strategies to counteract this problem could be a dietary supplementation capable of reducing protein degradability, thus improving ruminal activity, reducing environmental impact, and increasing the health value of animal products. The study was aimed at evaluating two varieties of faba beans, according to different polyphenol contents, in Cinisara grazing lactating cows for animal performances, grazing behaviour, and milk quality. A certain improvement in milk production was observed in cows supplemented with a high-polyphenol compared to a low-polyphenol faba bean variety and a soybean-based control group. Nevertheless, lower protein and casein proportions, together with higher milk urea nitrogen levels, were reported with a high polyphenols diet. The results seem to be in line with the hypothesis that tannin effects are not univocal but are dependent on several factors, such as basal diet composition, tannin dosage, and type.

## 1. Introduction

Legume grains (namely pulses) are being considered to be in competition between human and animal feeding; nonetheless, they could represent a valid option to support the protein requirements of livestock, replacing soybeans. Moreover, the ban of GMO soybean as a protein source in organic farming systems boosted the utilization of grain legumes in combination, as in low-input systems [1].

Among alternatives to soybeans, faba beans (*Vicia faba*), together with the protein pea (*Pisum sativum*), are the legume grains mainly used in animal nutrition. They are generally processed (through crushing or heat treatments) to inactivate potential antinutritional factors and then mixed to achieve the desired composition of the feed. Focusing on the nutritional composition of the faba bean (*Vicia faba*), its protein content is in the range of 21–34% on a DM basis [2,3,4], but it is relatively deficient in the sulphur amino acids, cysteine, and methionine [5]. In general, untreated legume seeds show a high crude protein degradability in the rumen that can be reduced through heat treatments [6,7]. Faba beans are also rich in starch content (30–43% on a DM basis), which also distinguishes them as an important energy source, either for ruminants or monogastrics [2]. The supplementation with 17% rolled or ground faba bean to replace soybean meal and corn grain in dairy cattle feed shows no detrimental effects on milk production, N excretion, and enteric CH_4_ production in dairy cows [8]. Also, the supplementation of 10% flaked faba to replace soybean meal and corn meal in the feed of Reggiana dairy cattle did not impair milk yield or composition [9].

One of the great challenges regarding pasture-grazing cows is a high intake of early-maturing herbage rich in highly soluble protein that could impair rumen efficiency [10]. The presence of a moderate level of polyphenolic substances in the diet of grazing animals could help to overcome this problem. Legume grains such as faba beans contain variable proportions of polyphenols such as tannins, particularly condensed tannins, mainly located in the seed coats (mean values reported in the literature ranged from 0.1 to 0.6 g/kg DM of condensed tannins), and high- and low-tannin varieties are available with differing nutritional values [2,11]. Among the strategies to manipulate the ruminal degradation of protein sources, with the aim to improve rumen undegradable protein (RUP), tannin supplementation is one of the potential options to increase protein flow to the duodenum, thus improving animal performance [12]. A comprehensive review clarified the scenario regarding the role of polyphenols in affecting gut function and health in ruminants, specifically focusing on their role in reducing protein degradability [13]. Nevertheless, tannin effects are not univocal, depending on basal diet, tannin type (hydrolysable or condensed), dietary concentration, the amount ingested, and animal species, according to their capacity to tolerate or degrade plant secondary compounds [14]. The use of a tannin-rich supplement could influence animal responses not only in terms of rumen efficiency but also in terms of feeding behaviour [15].

The aim of the study was to explore, in a Mediterranean semi-extensive system, the possibility of replacing dietary soy using a locally produced alternative feed that could potentially improve pasture utilization efficiency by providing an adequate protein level.

The effects of supplementation with two varieties of faba bean, with different polyphenol contents, were evaluated for milk yield and quality, as well as eating behaviour, in Cinisara grazing cows.

## 2. Materials and Methods

### 2.1. Animals and Diets

In a dairy cattle farm located in Piana degli Albanesi (a province of Palermo, Sicily), 30 Cinisara dairy cows were selected according to days in milk (DIM, 61 ± 29 days) and milk yield (12.9 ± 3.2 kg/head/day). The cows, fed for about 20 h/d on a mixed pasture (75% Gramineae, 15% legumes, 10% other species; initial biomass, 1.3 t DM/Ha; initial herbage height 12 cm) and supplied with 1 kg of a mixed hay (CP 10.2% DM) per day, were assigned, in a completely randomized design, to three dietary treatments and supplemented as follows: control group (C, 10 cows), 6 kg of grains mixture (an equal mixture of barley, oats, and wheat) and 0.4 kg soybean meal; low polyphenols group (LP, 10 cows), 4 kg of grains mixture and 2 kg/d of faba bean var. Torrelama (total polyphenols 4.4 mg GAE/g DM; CP 28.4% DM); high polyphenols group (HP, 10 cows), 4 kg of grains mixture and 2 kg/d of faba bean var. Fanfare (total polyphenols 16.4 mg GAE/g DM; CP 28.9% DM). Dietary supplementation was provided individually during the two daily milkings. The cows raised continuously grazed on 7 ha of natural pasture for 24 h/day, returning only for the two daily milkings. The cows were subjected to a pre-experimental period lasting 7 days, during which all the cows received a mixture of the three supplements, while the experimental period lasted 28 days.

### 2.2. Feedstuff Sampling and Analyses

Samples of different varieties of faba beans were previously collected, with the aim of identifying two varieties with low and high polyphenol contents. Sampling was carried out once, before the start of the trial, by taking three samples of each variety from three different containers and mixing them. The resulting single samples were subjected to analysis of total polyphenols. From the analysis of the content of total polyphenols, reported in the Table 1, it was highlighted that the varieties characterized by the lowest and highest levels of polyphenols were, respectively, Sikelia and Fanfare, which were then selected for the experiment.

Samples of pasture forage, hay, and experimental concentrates were collected weekly and pooled before analyses. Fresh pasture forage was frozen at −18 °C to be lyophilised. Feed analyses were performed according to AOAC [16] procedures for dry matter (DM, method 934.01) and crude protein (CP, method 2001.11). Neutral detergent fibre was determined using heat-stable amylase and exclusive of residual ash (aNDFom, method 2002.04), in accordance with the AOAC and Van Soest et al. [17].

The starch content was determined according to the method of Hall et al. [18].

Condensed tannins and total polyphenols were analysed as follows: 0.75 g powdered (<1 mm) feed samples were mixed with 25 mL acetone/water (70:30, *v*/*v*), sonicated for 30 min at 30 °C in an ultrasonic water bath (LBS1 Sonicator; Falc Instruments, Treviglio, Italy), and then centrifuged at 6000 rpm for 15 min at 4 °C; the supernatants were filtered through Whatman No. 541 filter paper and then stored at −18 °C until further analysis The butanol-HCl assay [19] was used to determine the CT in the extracts, expressed as delphinidin equivalent (g DE/kg DM) [20]; the sample absorbance was read at 550 nm in a HUCH DR3900 spectrophotometer (Hach, Loveland, CO, USA).

Extracts of samples were analysed for total polyphenols using the Folin–Ciocalteau colorimetric method [21]. In brief, 100 μL of each extract was transferred into a 15 mL centrifuge tube in which 900 μL distilled water and 500 μL Folin–Ciocalteau reagent, diluted with distilled water to a concentration of 1 N, were added. After 1 min, the mixture was supplemented with 2.5 mL of 20% (*w*/*v*) sodium carbonate, vortexed for 30 s, and incubated at room temperature in darkness for 40 min. The sample absorbance was read at 725 nm against a blank using a HUCH DR3900 spectrophotometer (Hach, Loveland, CO, USA). Aqueous solutions of different concentrations (0–1 mg/mL) of gallic acid were used to obtain the calibration curve (R^2^ = 0.99). The total polyphenols are expressed as a gallic acid equivalent (g GAE/kg DM).

### 2.3. Milk Sampling and Analyses

In the pre-experimental period and four times (every 7 days) during the experimental period, individual milk samples collected during the two daily milkings were used to compose weighted individual daily samples. These latter samples were analysed to determine lactose, fat, protein, casein, and urea levels using the infrared method (Combi-foss 6000, Foss Electric, Hillerød, Denmark).

The colour parameters of these milk samples were measured in duplicate with a Minolta Chroma Meter CR-300 (Minolta, Osaka, Japan) using illuminant C. The colour measurements are expressed as L* (lightness, from 0 = black to 100 = white), a* (redness, from green = −a to red = +a), and b* (yellowness, from blue = −b to yellow = +b), in accordance with the CIE L*a*b* system [22]. The same individual milk samples were also evaluated on the basis of their clotting ability using a Formagraph instrument (Foss Electric), measuring coagulation time (r, min), curd-firming time (k_20_, min), and curd firmness (a_30_, mm) in 10 mL of milk maintained at 35 °C and supplemented with 0.2 mL of a diluted solution (1.6:100) of rennet (1:15,000; Chr. Hansen, Parma, Italy). Fatty acids (FA) were directly methylated in lyophilised milk (100 mg) using 1 mL hexane with 2 mL 0.5 M NaOCH3 for 15 min at 50 °C, then supplemented with 1 mL of 5% HCl in methanol for 15 min at 50 °C, following the biomethylation procedure reported by Lee and Tweed [23]. The recovery of fatty acid methyl esters (FAME) was performed in 1.5 mL of hexane. Each sample (1 μL) was injected with an autosampler into a gas chromatography system equipped with a flame-ionisation detector (HP 6890, Agilent Technologies, Santa Clara, CA, USA). Sample FAME separation was achieved by a CP-Sil 88 capillary column (100 m long, 0.25 mm internal diameter, 0.25 μm film thickness) (Chrompack, Middelburg, The Netherlands). The temperatures of the injector and detector were kept at 255 °C and 250 °C, respectively; the flows of hydrogen, air, and helium were 40, 400 and 45 mL/min, respectively. The initial temperature of the oven was held at 70 °C for 1 min, increased by 5 °C/min up to 100 °C, held for 2 min, increased by 10 °C/min up to 175 °C, held for 40 min, then increased by 5 °C/min up to the final temperature of 225 °C and held for 45 min. The carrier gas was helium, used with a pressure of 158.6 kPa and a flow rate of 0.7 mL/min (linear velocity 14 cm/s). A FAME hexane mix solution (Nu-Check-Prep, Elysian, MN, USA) was employed for identification of each FA. Individual standards of odd and branched FAs, such as C15:0 iso, C15:0 anteiso, C17:0 iso, and C17:0 anteiso (Larodan Fine Chemicals AB, Malmö, Sweden), were used for their identification. A standard mixture of C18:2 c9 t11 (rumenic acid, RA) and C18:2 c10 t12 methyl esters (Sigma-Aldrich, Milano, Italy), together with published isomeric profiles, were used to identify the isomers of CLA [24,25]. As an internal standard to estimate total FAs, C23:0 (Sigma-Aldrich) was added in a dosage equal to 4 mg/g lyophilised milk.

The health value of the milk fat was estimated by calculating specific indexes, as follows.

Thrombogenic index (TI) = (C14:0 + C16:0 + C18:0)/(0.5 × MUFA + 0.5 × n-6 PUFA + 3 × n-3 PUFA + n-3/n-6) [26].

Atherogenic index (AI) = (C12:0 + 4 × C14:0 + C16:0)/(n-3 PUFA + n-6 PUFA + MUFA) [26].

### 2.4. Grazing Behaviour

The time dedicated to eat herbage was estimated twice during the experimental period, quantifying the sound of prehension activity during the 20 h of grazing, as follows: three cows per group were equipped with a collar with an audio recorder (Olympus (Tokyo, Japan) WS853) positioned under the neck for 24 h. During the first 2 h of grazing, the behaviour of each animal was recorded by visual observations. The behaviour was visually detected by six observers Each observer noticed the behaviour of three animals. In this way, each animal was followed by two observers (observer 1: cows 1, 2, 3; observer 2: cows 4, 5, 6; observer 3: cows 7, 8, 9; observer 4: cows 1, 4, 7; observer 5: cows 2, 5, 8; observer 6: cows 3, 6, 9). The visual detections were aimed at recognizing the moments during which the animals performed the activities of herbage prehension. This was in order to identify the correspondence between the observed activity and the related sound. Therefore, in two hours, hundreds of sounds of herbage prehension were detected, which allowed for the extension of the detection of the behaviour through listening to the recorded sounds from the entire grazing day.

Total eating time was measured, quantifying the time occupied in the activity of eating forage during grazing, exclusive of intra-meal intervals, but inclusive of biting and chewing, as defined by Gibb [27].

The number of bites per minute was also detected as follows: for each animal, during the main periods of eating activity, lasting more than 10 consecutive minutes, the number of bites taken in 1 min was detected for a number of times proportional to the duration of the single eating activity (from 10 to 30 times).

### 2.5. Statistical Analysis

The data relating to milk production and the chemical–physical characteristics of the milk and cheese were subjected to GLM analysis, with repeated measures to test the effect of dietary treatment. The pre-treatment data were used as covariates. When the covariate was not significant, (*p* > 0.05) it was excluded from the model. Mean eating time and number of bites per minute were analysed using the univariate general linear model.

Significant differences were considered when *p* < 0.05, while a significant tendency was identified when 0.05 > *p* < 0.10. The LSD test was used to compare the groups means.

The analysis was performed using the SPSS 26 statistical package.

## 3. Results

Supplemented diets were totally ingested, without orts. The HP- and LP-supplemented diets showed higher protein contents and less NDF compared to those of group C. As expected, the HP supplement was richer in total polyphenols and condensed tannins compared to the other diets (Table 2).

In Table 3, the milk yield and composition and feeding behaviours at pasture are reported.

The milk yield was not statistically (*p* ≤ 0.05) affected by the treatments, although a trend (*p* = 0.057) was evident towards a higher production level in the HP group compared to the C group. FPCM was not affected by treatment. The HP cows showed lower milk protein (*p* = 0.009) and casein (*p* = 0.007) levels compared to those of their counterparts. No significant differences in lactose levels were found among the dietary treatments. The urea level in HP milk was significantly higher (*p* = 0.036) compared to that in C milk, while an intermediate level was measured in LP milk. Moreover a tendency toward an increase of milk urea in the LP group compared to that in the C group was observed.

Protein (*p* = 0.053), casein (*p* = 0.061), and lactose (*p* = 0.051) yields in the HP group showed a tendency toward increased levels compared to those in the C group.

Treatment did not affect the eating time or the biting rate.

In Table 4, the milk fatty acid profile is reported. No significant differences occurred related to total SFA, MUFA, PUFA, UFA, BCFA, UFA/SFA ratio, total CLA, PUFA/SFA ratio, n-6, or n-3. The linoleic (LA) to alfa-linolenic (ALA) ratio was lower (*p* < 0.05) in HP milk compared to LP milk, while an intermediate value was reported in C milk. TI and AI were not affected by dietary treatments.

In Table 5, the coagulation properties and colour parameters of the milk used for micro cheese-making are reported. The dietary treatments did not affect any of the rennet coagulation properties or the colour coordinates.

## 4. Discussion

One of the main goals for improving livestock production sustainability is to increase protein source self-sufficiency and/or explore novel protein sources as alternatives to soybeans, largely imported in Europe and challenging in terms of environmental impact [28]. Legume seeds, particularly the faba bean, which is one of the oldest crops cultivated worldwide, can play a role in this regard [29]. In our trial, the chemical composition of the selected faba bean varieties exhibited different polyphenol contents, which could have affected some findings. The variety named “Fanfare” showed a higher polyphenol and total tannin contents compared to those of the “Sikelia” variety.

The different polyphenol and condensed tannin (CT) contents tended to affect milk yield, which was higher in cattle fed the HP diet compared to that of those fed the LP and C diets. This could be due to a probable reduced protein degradability at the rumen level, usually occurring at a high rate in faba beans [30], which could have increased the protein flow to the small intestine, reducing nitrogen loss from the rumen. It is noteworthy that polyphenols, and tannins in particular, at low to moderate concentrations protect dietary protein against ruminal degradation [11]. A literature meta-analysis on the effects of dietary tannins on milk yield and composition, as well as on the efficiency of nitrogen use in dairy cows, suggested an absence of tannin effects on corrected milk. However, a greater milk yield is observed when cows are fed forage or by-products naturally rich in tannins than when they receive tannin extracts, but the effects of the dose or type of tannins (hydrolysable vs. condensed) are still controversial [31].

Under our conditions, milk protein and casein percentages were lower in HP milk compared to those in LP and C milk. It is probable that a limited microbial protein synthesis occurred in the rumen, thus reducing amino acids availability in the small intestine. Indeed, in terms of milk yield components, expressed as g/d, the HP diet tended to improve protein and casein levels compared to those in its counterparts, thus justifying a certain effect of high polyphenol dietary contents, probably linked to better dietary protein utilization.

Milk urea nitrogen (MUN) levels were higher in HP milk compared to those in C milk, while LP milk showed an intermediate value. The literature is quite consistent in emphasizing the effect of tannins from the diet in decreasing the levels of urea in milk [31]. Generally, high levels of MUN can be an indicator of dietary degradable protein excess in the diet [32] or a deficiency of energy [33]. Nonetheless, the presence of tannin in the faba bean does not seem to protect nitrogen from degradability, according to the results reported by Foucher et al. (unpublished), cited by Crépon et al. [2].

The faba bean-supplemented diets supplied higher crude protein and lower starch levels compared to those in the C diet; this could also justify the unexpectedly higher MUN content in these groups. In any case, an excess of urea in milk should have created problems in cheese making, while under our conditions, no significant differences in the coagulation parameters were detected.

Different CT dosages are thought to affect animal performance, but it is also noteworthy that the same concentration of condensed tannins could drive different outcomes, according to the CT sources [34]. The literature reports a reduction in ruminal protein degradation, with a greater amino acid absorption in the hind gut, when the intake of CT is under 50 g CT/kg DM [35].

In our experiment, the CT levels of the HP diet (8.1 g DE/kg DM) probably did not alter protein metabolism towards a reduction in milk urea nitrogen.

**Figure 1:**
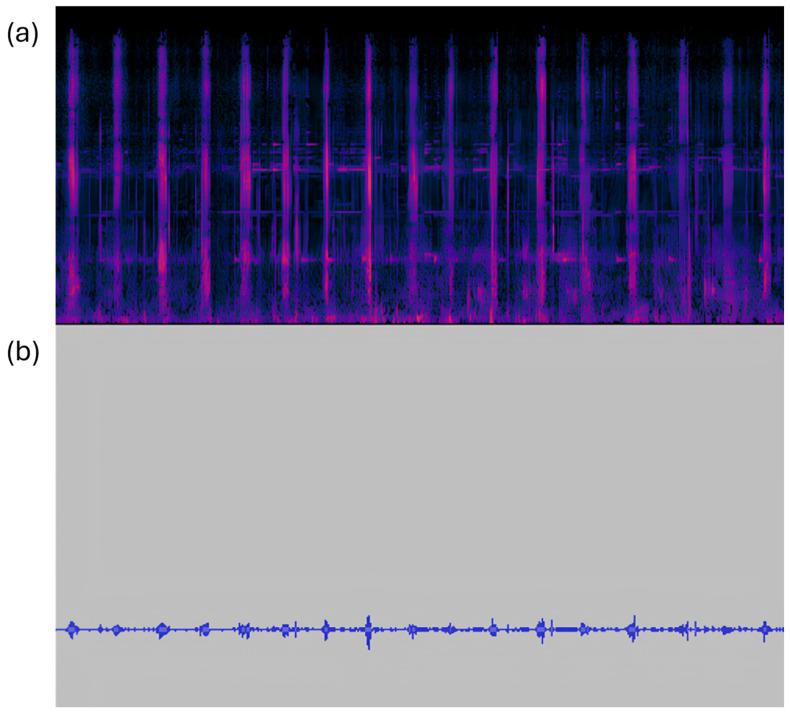
Audio recording of cow grazing activity. (**a**) Spectrogram of the recorded bites, showing the signal intensity across frequency and time; distinct bands highlight the spectrum profile of each bite. (**b**) Waveform of the recording, with peaks corresponding to each biting event.

Another hypothesis supporting the different urea levels between groups could have been a variation in herbage intake, whose protein degradability is well known [36]. However, this does not appear to have occurred in our trial. Under our conditions, although data on intake at pasture were not available, the grazing sounds were recorded, allowing us to estimate the eating time and the number of bites/minute. The bite sound was highly recognizable and easily identifiable [37,38], both on the basis of the sound emitted by the tearing of herbage and by observing the typical sound wave form and spectrum profile (Figure 1). These two variables were not significantly affected by the treatments. It is well known that herbage intake represents the product of eating time x the number of bites x the bite mass [39]. Considering that bite mass is strictly related to pasture characteristics (height, biomass) [40], it is possible to hypothesize that the intake component, under our conditions, was similar for the three groups, since they grazed on the same pasture. Therefore, it seems possible to assume that herbage intake was not influenced by the three supplements. Similarly, Araujo et al. [41] did not find any grazing behaviour variation that supplied grazing cows with tannins. Vilas Boas Fonseca et al. [42], supplying energy feedstuff containing condensed tannin (CT) to grazing beef cattle, reached similar DMI levels between groups.

Dietary treatments did not significantly change the milk fatty acid profile. Only the linoleic (LA) to alfa-linolenic (ALA) ratio was lower in the HP compared to that in the LP group. Among polyphenol compounds, tannins can play a role in modulating fatty acids composition in ruminant-derived products. Nevertheless, the tannin effects were controversial, according to the different tannin types, dosage, and interaction with other dietary ingredients [14]. Generally, tannins tend to protect dietary PUFAs from biohydrogenation occurring in the rumen, improving their presence in ruminant products, as well as modifying the concentration of some BH intermediates. In particular, there is evidence that tannins could be a useful tool to increase the accumulation of C18:1 t11 VA in the rumen and promote the endogenous synthesis of C18:2 c9 t11 (CLA or rumenic acid). In our experiment, no differences among groups were detected for either vaccenic or CLA fatty acids in milk, probably due to the nature of polyphenols of the faba bean varieties that did not affect the rumen microbiota responsible for the different BH steps. The literature also reveals controversial results regarding the effect of tannins in modulating fatty acid BH, as reported by Frutos et al. [14].

Moreover, the milk rennet properties were not affected by dietary treatments, despite the differences among groups in regards to casein proportions, in line with the findings of Volpelli et al. [5].

## 5. Conclusions

The experimental trial was designed to test whether the use of faba bean varieties with different polyphenol and condensed tannin contents (high vs. low) in diets offered to grazing, low-producing Cinisara cows could drive outcomes in terms of improving milk quality or modifying grazing behaviour.

The results do not seem to suggest a different efficiency of use of the supplements with different polyphenol contents nor a different pasture utilization.

The main findings of the trial include a certain improvement in milk production in cows fed a high-polyphenol faba bean diet (which provided 0.42 g polyphenols/kg LW/d) compared to that of the control diet, along with lower protein and casein proportions, along with a higher milk urea nitrogen level. The latter result seems to be coherent with the hypothesis that tannin effects are influenced by the tannin dosage and types, which could not lead to a univocal output.

In our trial, a supplement including a faba bean variety with a high polyphenol (condensed tannins) content did not change the milk coagulation properties, which is important due to the nearly exclusive use of Cinisara milk for cheese making. A slight improvement in the milk fatty acid profile in terms of a low LA to ALA ratio led to an effect of HP supplementation in terms of the healthiness of milk. Therefore, the choice of supplementation with faba beans with different polyphenol contents for grazing cows should be calibrated to the pasture characteristics and feed intake, as well as to the processing/not processing of legumes (heat treatment) before supplementation. More research is needed to clarify these points.

The results would seem to demonstrate that the use of faba beans, regardless of their polyphenols content, could represent an excellent alternative to soyabeans, but heat treatment is probably suggested to reduce protein degradability in the rumen, which could impair rumen efficiency. Nevertheless using a faba bean variety rich in polyphenols as a pasture supplement slighty increased milk production, without significantly degrading the qualitative characteristics of the milk. Likely, under our conditions, the CT dosage in the HP supplement and the relative intake did not lead to a strong effect in terms of improving protein metabolism, with an improvement in amino acid absorption in the small intestine, thus confirming once more the controversial results reported in the literature according to a number of variables, such as CT dosage, basal diet characteristics, amount ingested, and animal species.

## Figures and Tables

**Table 1:** Analysis of content in total polyphenols of different varieties of faba beans.

Varieties	Total Polyphenols g GAE ^a^/kg DM
Torre Lama brown (Villarosa, Enna, Italy)	10.5
Sicania	9.6
Sikelia	4.4
Fanfare	16.4
Torre Lama brown (Licata, Agrigento, Italy)	8.7
Torre Lama brown (Piana degli Albanesi, Palermo, Italy)	8.3

^a^ GAE, gallic acid equivalent.

**Table 2:** Mean chemical composition of pasture forage and supplements ^a^.

	Pasture	Hay	C	HP	LP
DM%	22.5	90.2	89.2	89.4	89.4
Crude protein (CP)% DM	17.7	10.2	15.7	18.5	18.3
^b^ aNDFom% DM	40.4	50	49.2	44.2	45.6
Starch% DM	1.2	2.3	53.9	51.8	51.5
Total polyphenols ^c^ GAEg/kg DM	12.1	10.3	4.2	8.3	4.3
Condensed tannins ^d^ gDE/kg DM	3.2	3.8	0.72	8.1	1.3

^a^: C, control; HP, high polyphenols; LP, low polyphenols; ^b^ aNDFom, neutral detergent fibre using heat-stable amylase and exclusive of residual ash; ^c^ GAE, gallic acid equivalent; ^d^ DE, delphinidin equivalent.

**Table 3:** The effects of the dietary treatments ^c^ on grazing behaviour, milk yield, and milk composition.

	C	HP	LP	SEM	*p*-Value
Eating time at pasture (min/d)	423	467	431	16.8	0.549
Biting rate (n. bites/min)	48	40	38	2.21	0.165
Milk yield, kg/d	14.6 ^Aa^	17.1 ^Ab^	15.3 ^Aab^	0.62	0.057
FPCM ^d^ kg/d	11.7	12.6	11.4	0.535	0.385
Fat%	3.19	2.93	2.99	0.08	0.143
Protein%	3.47 ^Bb^	3.20 ^Aa^	3.39 ^Bb^	0.04	0.009
Lactose%	4.80	4.86	4.85	0.03	0.130
Casein%	2.74 ^Bb^	2.45 ^Aa^	2.67 ^Bb^	0.03	0.007
Urea mg/dL	20.9 ^Aa^	25.5 ^Bb^	22.9 ^ABb^	1.07	0.036
Fat g/d	465.0	508.0	453.8	19.2	0.296
Protein g/d	496.3 ^Aa^	554.9 ^Ab^	517.7 ^Aab^	17.5	0.053
Lactose g/d	702.9 ^Aa^	829.6 ^Ab^	743.4 ^Aab^	30.2	0.051
Casein g/d	388.6 ^Aa^	432.8 ^Ab^	410.9 ^Aab^	13.3	0.061

^A,B^: Different superscripts within a row indicate significant differences (*p* < 0.05); ^a,b^: different superscripts within a row indicate a statistical tendency (*p* < 0.10); ^c^: C, control; HP, high polyphenols; LP, low polyphenols; ^d^: fat- and protein-corrected milk.

**Table 4:** Milk fatty acid composition (% total FA) as affected by dietary treatments ^c^.

	C	HP	LP	SEM	*p*-Value
SFA ^d^	68.1	68.7	68.0	0.57	0.888
MUFA ^e^	26.8	26.1	26.9	0.49	0.779
PUFA ^f^	4.91	4.88	5.01	0.13	0.903
UFA ^g^	31,7	30.9	31.9	0.60	0.810
BCFA ^h^	2.58	2.50	2.60	0.04	0.601
UFA/SFA	0.47	0.45	0.48	0.01	0.770
Total CLA ^i^	1.60	1.51	1.58	0.07	0.818
PUFA/SFA	0.073	0.071	0.075	0.001	0.824
n-6	2.39	2.36	2.44	0.06	0.869
n-3	0.91	1.01	0.99	0.03	0.344
n-6/n-3	2.72 ^Bb^	2.40 ^Aa^	2.54 ^ABab^	0.07	0.077
LA ^l^/ALA ^m^	1.46 ^ABb^	1.31 ^Aa^	1.51 ^Bb^	0.04	0.046
TI ^n^	2.83	2.79	2.80	0.07	0.952
AI ^o^	2.67	2.77	2.78	0.08	0.816

^A,B^: Different superscripts within a row indicate significant differences (*p* < 0.05); ^a,b^: different superscripts within a row indicate a statistical tendency (*p* < 0.10); ^c^: C, control; HP, high polyphenols; LP, low polyphenols; ^d^: SFA, saturated fatty acids; ^e^: MUFA, monounsaturated fatty acids; ^f^: PUFA, polyunsaturated fatty acids; ^g^: UFA, unsaturated fatty acids; ^h^: BCFA, branched-chain fatty acids; ^i^: CLA, conjugated linoleic acid; ^l^: LA, linoleic acid; ^m^: ALA, alfa-linolenic acid; ^n^: TI, thrombogenic index; ^o^: AI, atherogenic index.

**Table 5:** Rennet coagulation properties and colour parameters of milk used for micro cheese-making as affected by dietary treatments ^a^.

	C	HP	LP	SEM	*p*-Value
Rennet coagulation properties					
Curd firmness, A_30_ (mm)	30.0	26.7	29.0	1.10	0.357
Curd firming time, K_20_ (min)	6.46	7.22	6.77	0.19	0.146
Coagulation time, r (min)	22.5	24.0	22.7	0.61	0.506
Colour coordinates					
Lightness, L*	88.5	87.8	87.7	0.47	0.119
Redness, a*	−4.80	−4.74	−4.81	0.07	0.903
Yellowness, b*	11.4	10.5	11.3	0.38	0.410

^a^: C, control; HP, high polyphenols; LP, low polyphenols.

## Data Availability

The data are contained in the article.
